# The Impact of Cataract Surgery on Activities and Time-Use: Results from a Longitudinal Study in Kenya, Bangladesh and the Philippines

**DOI:** 10.1371/journal.pone.0010913

**Published:** 2010-06-01

**Authors:** Sarah Polack, Christina Eusebio, Wanjiku Mathenge, Zazkia Wadud, Mamunur Rashid, Allen Foster, Hannah Kuper

**Affiliations:** 1 Department of Infectious and Tropical Diseases, London School of Hygiene and Tropical Medicine, Keppel Street, London, United Kingdom; 2 Cataract Foundation of the Philippines, Bacolod, Philippines; 3 Eye Unit, Rift Valley Provincial Hospital, Nakuru, Kenya; 4 Child Sight Foundation, Dhaka, Bangladesh; 5 CSS Rawm Hospital, Khulna, Bangladesh; Tulane University, United States of America

## Abstract

**Background:**

Cataract is the leading cause of blindness in the world, and blindness from cataract is particularly common in low-income countries. The aim of this study is to explore the impact of cataract surgery on daily activities and time-use in Kenya, Bangladesh and the Philippines.

**Methods/Principal Findings:**

A multi-centre intervention study was conducted in three countries. Time-use data were collected through interview from cases aged ≥50 years with visually impairing cataract (VA <6/24) and age- and gender-matched controls with normal vision (VA≥6/18). Cases were offered free/subsidized cataract surgery. Approximately one year later participants were re-interviewed about time-use. At baseline across the three countries there were 651 cases and 571 controls. Fifty-five percent of cases accepted surgery. Response rate at follow up was 84% (303 out of 361) for operated cases, and 80% (459 out of 571) for controls. At baseline, cases were less likely to carry out and spent less time on productive activities (paid and non-paid work) and spent more time in “inactivity” compared to controls. Approximately one year after cataract surgery, operated cases were more likely to undertake productive activities compared to baseline (Kenya from 55% to 88%; Bangladesh 60% to 95% and Philippines 81% to 94%, p<0.001) and mean time spent on productive activities increased by one-two hours in each setting (p<0.001). Time spent in “inactivity” in Kenya and Bangladesh decreased by approximately two hours (p<0.001). Frequency of reported assistance with activities was more than halved in each setting (p<0.001).

**Conclusions/Significance:**

The empirical evidence provided by this study of increased time spent on productive activities, reduced time in inactivity and reduced assistance following cataract surgery among older adults in low-income settings has positive implications for well-being and inclusion, and supports arguments of economic benefit at the household level from cataract surgery.

## Introduction

Cataract is the leading cause of blindness and low vision worldwide[1]. The burden of visual impairment from cataract is particularly high in the poorest countries, where it is commonly responsible for at least 50% of all blindness[2]. Visual impairment from cataract is associated with increased difficulties with activities of daily living and reduced quality of life.[3]–[6]

Engagement in leisure and productive activities (paid and non-paid e.g. domestic activities, home food production, child-care) is an important component of well-being in later life. [7]–[8] Productive activities are also likely to contribute to individual and household economy.[9] In rural low-income settings, where the prevalence of un-operated cataract is highest, all household members regardless of age commonly contribute to the basic needs of a household [10]. While direct employment opportunities may be limited in these settings, particularly in the age group most affected by cataract, the economic contribution of non-paid productive activities is well recognised. [9]

A positive effect of increased engagement in daily activities following cataract surgery is frequently asserted, yet there is a lack of empirical evidence either supporting or refuting this. Previous studies have shown using visual functioning questionnaires that people report less difficulty undertaking daily activities after cataract surgery.[11]–[14]. However, information is lacking on whether actual participation in and time spent on different daily activities changes following cataract surgery. No prospective studies comparing time-use patterns before and after cataract surgery could be identified. This link is likely to be complicated because cataract predominantly affects older people who may experience other co-morbidities influencing their engagement in activities.

The ‘Cataract Impact Study’ was undertaken to explore the impact of cataract surgery on poverty, quality of life and time-use in Kenya, Bangladesh and the Philippines among people aged ≥50 years. At baseline we found that people visually impaired from cataract (cases) were less likely to participate in and spent less time on productive activities compared to age- and gender-matched controls with normal vision.[15] Cases also spent more time in ‘inactivity’ and were more likely to require assistance with activities. At baseline, cases were offered cataract surgery and were followed up after one year. The aim of the current paper is to assess the impact of cataract surgery by comparing participation, time spent and assistance with different daily activities before and one year after cataract surgery in these three different low-income settings.

## Methods

Ethical approval for this study was granted by ethics committees of the London School of Hygiene &Tropical Medicine, Kenya Medical Research Institute, Bangladesh Medical Research Council and the University of St. La Salle, Bacolod, The Philippines. Informed signed/thumb-printed consent was obtained from all study participants.

### Study overview

The ‘Cataract Impact Study’ was a longitudinal intervention study conducted in Kenya (Nakuru district), Bangladesh (Satkhira district) and the Philippines (Negros Island and Antique district). At baseline cases with visual impairment from cataract and controls without visual impairment were identified and interviewed about time-use, health related quality of life and poverty. All cases were offered free or subsidized surgery. Approximately one year later cases and controls were re-traced, re-examined and re-interviewed. This paper presents the findings from the time-use data.

### Study population

Sample size calculations were powered to detect a 30% increase in time spent on productive activities, with an alpha of 0.05 and 80% power. To detect this improvement required 100 operated cases in each country examined at baseline and follow-up.

Cases and controls were identified primarily through population-based surveys of blindness which included >3600 people aged ≥50 years in each setting.[16] Clusters were selected using systematic cluster sampling with probability proportionate to size and within clusters, households were selected through compact segment sampling.[17] People identified in the survey aged ≥50 years with pinhole corrected visual acuity (VA) <6/24 in the better eye due to cataract were eligible to be cases. VA was measured using a tumbling E chart and the presence of cataract was assessed through ophthalmic examination with a direct ophthalmoscope. One (or up to two in Bangladesh) age- gender and cluster matched controls without visual impairment from cataract were selected per case identified in the survey. During the survey the eligible controls in each cluster were listed by gender and age group (50–54, 55–59, 65–69 and ≥70 years). When a case was identified, one control (or up to two in Bangladesh) of the same gender and age group were selected at random for inclusion. If there were no matching controls at that time then the subsequent eligible control identified in the cluster was recruited.

Due to logistical and time constraints, additional cases were identified in each setting through community-based case detection. In Kenya and Negros (Philippines) additional clusters were selected using probability proportionate to size sampling after completion of the population based-survey. These clusters were visited in advance and asked that all people aged ≥50 years with eyesight problems come to a central point on a specified day and that people unable to attend (e.g. due to blindness or physical disability) be noted. All people attending the central point and those unable to leave their households underwent an eye examination using the procedures described above. People who met the case definition were invited to participate in the study and were interviewed in their homes. In Bangladesh and Antique (Philippines), case finding was conducted simultaneously with the survey so that age- gender matched controls were also included for these cases. In each cluster the teams asked to be taken to a community member with eye problems living within the boundaries of the cluster but outside the selected segment. The ophthalmologist conducted the ophthalmic examination at the household to identify eligible cases. In Kenya the first 50 patients (from a set date) attending the local hospital for cataract surgery and meeting the case definition were also recruited for the study.

### Baseline and follow up

Baseline surveys were conducted between Jan 2005-May 2006. All cases were counselled and offered surgery at one hospital in the study district. In Kenya and Bangladesh free surgery was offered. In the Philippines a fee was requested but those who could not afford the fee were offered free surgery. Follow up surveys were undertaken approximately one year later, during the same climatic season. Interviews were conducted in respondents' own homes (except for hospital cases in Kenya) by trained interviewers who were regularly observed by supervisors. The same interviewers were involved at baseline and follow up, except in Bangladesh where two additional people were trained at follow up. Visual acuity of cases and controls at follow up was assessed using the same procedure as baseline.

### Measuring time-use

Time-use data were collected using the ‘stylised activity list’ developed for the World Bank's Living Standards Measurement Survey.[10] Participants were asked whether they had been involved in each of a preset list of common daily activities during the last week and if yes, if they had been involved in the activity yesterday. Those who had been involved in an activity ‘yesterday’ were asked to estimate how much time they had spent and whether they received any assistance from another person in performing that activity. Interviewers were instructed to check that total time reported was 22–26 hours and if not, to go through the list again with the respondent. Activities were grouped as follows:

- Personal: Sleep, bathing, dressing, eating, other
- Household/family: Cooking/washing dishes, cleaning house/clothes, shopping, looking after children/elderly/sick, other
- Paid work: paid employment, commission work, self employed/own business, other
- Work for own use: Agriculture, animal rearing, fetching firewood/water, processing agricultural products/food, other
- Leisure outside home: social visits, attending ceremonies, attending meetings
- Leisure inside home: reading/listening to radio/watching TV; chatting, relaxing with friends/family; prayer (in Bangladesh), other
- No activity
- Travel

Household/family work, work for own use and paid work were all defined as productive activities.

### Covariates

Data were collected on standard socio-demographic indicators and a socio-economic-status (SES) index for each household was developed using Principal Components Analysis, separately for each country.[18]

### Statistical analysis

For the purposes of these analyses only controls with VA≥6/18 were included. The mean proportion of time spent on each activity group was calculated by dividing total minutes on specific activity group by the sum of minutes reported on all activities for that individual (as not all totals were exactly 24 hours). To ease interpretation, we then converted the proportion of time into hours and minutes (e.g. if the proportion of reported time on household work was 25%, this was presented as 6hours). All eligible respondents were included in this calculation whether or not they had participated in that activity. Participants were excluded from the analysis of time spent on activities if the total reported time spent on all activities was <19 hours or >30 hours.

We assessed changes in time-use following cataract surgery by comparing time-use at baseline and follow up restricted to participants who had data at both time points. McNemars test was used to compare engagement in activities during the last week (binary variable) among operated cases, un-operated cases and controls (separately). Average time spent on each activity ‘yesterday’ (continuous variable) was compared for each group using confidence intervals and paired t-tests. Since a substantial number of participants did not participate in household work, work for own use, paid work and leisure outside the home during the previous day, we calculated bootstrap confidence intervals for these variables and the statistical comparison of baseline and follow up time use was repeated using Wilcoxon signed rank test. Findings were essentially unchanged and therefore parametric results are presented. Case/Control comparisons were controlled for the matching variables (age and gender) and SES. Multivariate linear regression analyses were undertaken to explore socio-demographic and ocular (baseline and follow up VA) predictors of change in time spent on productive activities and ‘inactivity’ among operated cases. For these analyses the data for all three countries were combined. This was considered acceptable because consistent trends in change in time-use were observed between the three settings.

## Results

At baseline we included 196 cases visually impaired from cataract in Kenya and 128 controls with normal vision, 217 cases and 280 controls in Bangladesh, and 238 cases and 163 controls in Philippines. All controls and the majority of cases (n = 389, 60%) were identified through the survey, rather than case finding (n = 213, 33%) or from the hospital (n = 49, 8%, Kenya only). Sixty-eight percent of cases (n = 132) identified at baseline attended for surgery in Kenya, 46% (n = 117) in Bangladesh and 47% (n = 112) in the Philippines (“operated cases”).

Overall response rate at follow-up was high. In Kenya we re-examined 80% (n = 106) of the operated cases, 65% (n = 40) of un-operated cases and 75% (n = 96) of controls (table 1). Response rates were similar in Bangladesh (85%, 71%, 80%) and the Philippines (88%, 73%, 86%). The causes of non-response were death or the person not available. Refusals to participate at follow up were rare (2 participants in Kenya, 3 in Bangladesh and 3 in the Philippines). Operated cases and controls lost to follow up did not differ systematically from those included in terms of socio-demographic characteristics (data not presented). Operated cases lost to follow up were slightly more likely to participate in productive activities at baseline compared to the operated cases included at follow-up, reaching statistical significance only in the Philippines (data not presented). The final analyses included 106 operated cases and 96 controls in Kenya, 99 operated cases and 223 controls in Bangladesh and 98 operated cases and 140 controls in the Philippines. Among the operated cases at follow up, 71% in Kenya and 72% in the Philippines had presenting VA ≥6/18 in the better eye while this was higher in Bangladesh (84%).

**Table 1:** Loss to follow up statistics by country for operated cases, un-operated cases and controls.

Country	Participant type	Total at Baseline N	Examined at follow up N (%)	Lost to follow-up N (%)
Kenya	Operated cases	132	106 (80%)	26 (20%)
	Controls	128	96 (75%)	32 (25%)
	Un-operated cases	62	40 (65%)	22 (35%)
Bangladesh	Operated cases	117	99 (85%)	18 (15%)
	Controls	280	223 (80%)	57 (20%)
	Un-operated cases	100	71 (71%)	29 (29%)
Philippines	Operated cases	112	98 (88%)	14 (12%)
	Controls	163	140 (86%)	23 (14%)
	Un-operated cases	126	92 (73%)	34 (27%)

Operated cases and controls were similar in age, gender and marital status but in Kenya and Bangladesh operated cases were less likely to have had a formal education or be in the highest SES group compared to controls (table 2). Un-operated cases were significantly older than operated cases in Kenya and the Philippines and were less likely to be married in Kenya and Bangladesh (data not presented). In Kenya un-operated cases had better vision at baseline compared to operated cases but in the other two countries the baseline VA distribution was similar between operated and un-operated cases.

**Table 2:** Demographic and visual acuity characteristics of operated cases and controls examined at baseline and follow-up.

	Kenya			Bangladesh			The Philippines		
	Operated cases (n = 106)	Controls (n = 96)	p-value*	Operated cases (n = 99)	Controls (n = 223)	p-value*	Operated cases (n = 98)	Controls (n = 140)	p-value*
**Mean age (95% CI)**	74 (72–76)	73 (71–75)	0.7	70 (68–72)	68 (67–69)	0.1	71 (69–73)	70 (69–72)	0.6
**Female**	56%	56%	0.9	53%	57%	0.4	61%	54%	0.2
**Married**	52%	58%	0.4	49%	58%	0.1	44%	57%	0.04
**Education**	25%	45%	0.004	18%	34%	0.04	90%	92%	0.5
**Highest SES** ±	18%	38%	0.002	16%	29%	0.01	18%	29%	0.07
**Baseline VA** ¥									
≥6/18	0%	100%		0%	100%		0%	100%	
<6/24–6/60	39%			29%			23%		
<6/60–3/60	17%			16%			22%		
<3/60>PL	21%			12%			28%		
PL	24%			42%			28%		
**Follow up VA** †									
≥6/18	71%			84%			72%		
<6-18–6/60	21%			6%			20%		
<6/60	8%			10%			8%		
**Surgery**	57%			74%			71%		
Unilateral	43%			26%			29%		
Bilateral									

*P-value from anova (continuous variables) or chi Square (categorical variables) comparing operated cases and controls.

± Highest quartile of socio-economic status (SES).

¥Presenting visual acuity in the better eye at baseline.

† Presenting visual acuity in the better eye among operated cases at follow up classified by WHO cataract surgical outcome categories.

PL, Perception of light.

### Changes in time-use after cataract surgery

- i) Activities carried out during previous week
  At baseline in each country cases were significantly less likely than controls to undertake productive activities (Kenya, odds ratio (OR) 0.2, 95% Confidence Intervals (CI) 0.1–0.3; Bangladesh OR 0.1, 95% CI 0.1–0.2; Philippines OR 0.3 95% CI 0.2–0.6). At follow-up, the proportion of operated cases that carried out productive activities had increased significantly (table 3). Overall, nearly 25% more operated cases undertook productive work at follow-up in Kenya and Bangladesh, while for the Philippines the increase was 10% (reflecting the higher proportion engaged at baseline). In contrast, among controls, engagement in productive activities remained similar at baseline and follow up.
  In each country there was an increase of at least 20% in the proportion of operated cases participating in leisure outside of the household (p<0.005). However this trend was also observed among controls in the Philippines and Bangladesh.
- ii) Time spent on activities during previous day
  The proportion of outliers excluded from these analyses was 6% in Kenya (<19 hours 4%, >29 hours 2%), 0.4% Bangladesh (<19 hours 0.2%, >29 hours 0.2%) and 12% in The Philippines (<19 hours10%, >29 hours 2%).

**Table 3:** Activities carried out (during previous week) and assistance with activities (previous day) among operated cases and controls at follow up compared to baseline in Kenya, Bangladesh and the Philippines.

		Kenya			Bangladesh			Philippines		
Activity group	Case/control	Baseline N (%)	Follow up N (%)	p-value*	Baseline N (%)	Follow up N (%)	p-value*	Baseline N (%)	Follow up N (%)	p-value*
Productive activities										
	Operated cases	53 (52%)	76 (75%)	<0.001	59 (60%)	82 (83%)	<0.001	79 (81%)	89 (91%)	0.01
	Controls	81 (88%)	75 (82%)	0.2	211 (95%)	204 (92%)	0.1	132 (94%)	131 (94%)	0.9
	*p-value:* cases vs controls†	<0.001	0.4		<0.001	0.02		0.001	0.2	
Leisure outside household										
	Operated cases	34 (33%)	58 (59%)	<0.001	37 (37%)	69 (70%)	<0.001	37 (38%)	56 (57%)	0.005
	Controls	56 (61%)	58 (63%)	0.8	91 (41%)	69 (70%)	<0.001	62 (44%)	103 (74%)	0.001
	p-value: cases vs controls†	<0.001	0.2		0.6	0.5		0.3	0.02	
Leisure inside household										
	Operated cases	81 (79%)	85 (83%)	0.4	98 (99%)	99 (99%)	N/A	91 (93%)	95 (97%)	0.2
	Controls	79 (86%)	82 (89%)	0.4	222 (99%)	222 (99%)	N/A	133 (95%)	138 (99%)	0.06
	p-value: cases vs controls†	0.2	0.2		0.6	0.5		0.5	0.7	
Assistance with any activity										
	Operated	26 (25%)	12 (12%)	0.01	43 (43%)	19 (19%)	<0.001	23 (23%)	1 (1%)	<0.001
	Controls	2 (2%)	6 (7%)	0.2	20 (9%)	35 (16%)	0.03	12 (9%)	7 (5%)	0.2
	p-value: cases vs controls†	<0.001	0.2		<0.001	0.4		0.02	<0.001	

*P-value from McNemars chi squared test comparing participation at baseline and follow up among i) operated cases and ii) controls.

†P-value from chi square comparing participation between operated cases and controls at i) baseline and ii) follow up.

NB Data are not presented for inactivity as nearly all (>97%) cases and controls engaged in this at baseline and follow up.

#### Productive activities

**Figure 1:**
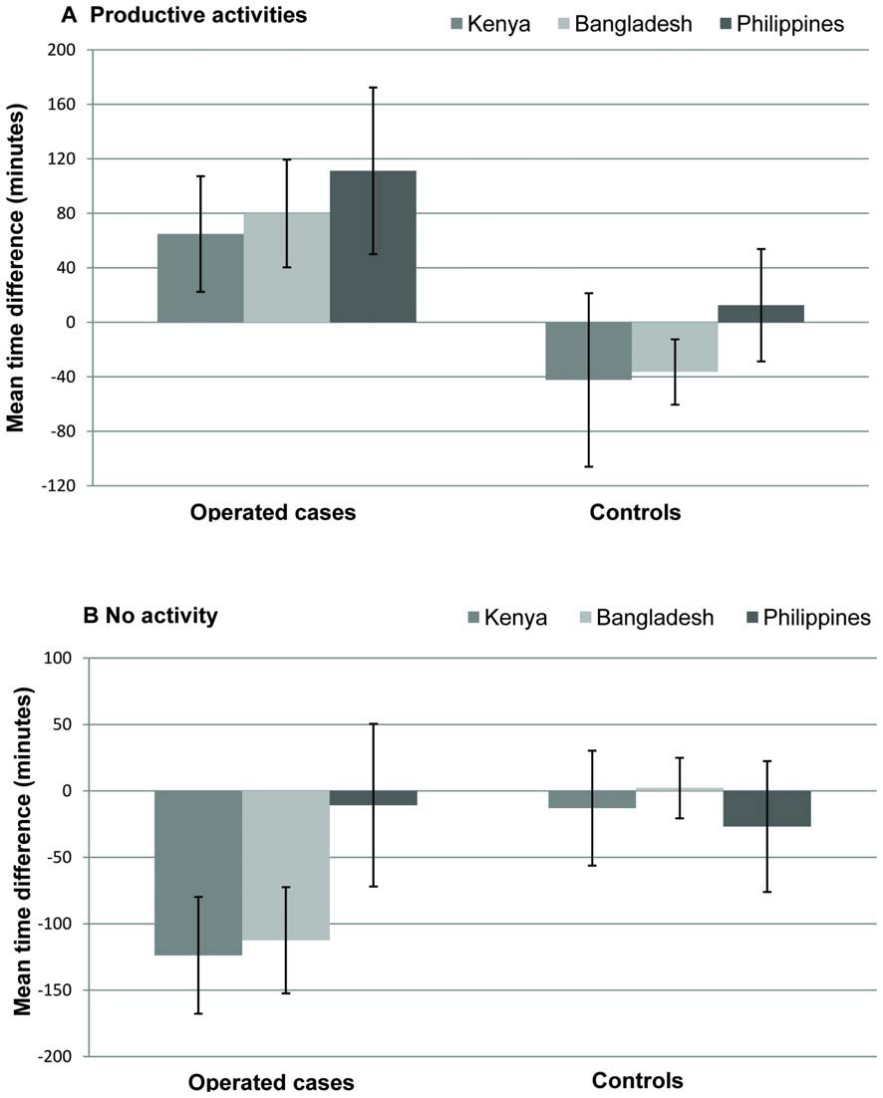
Change in time spent on productive activities and inactivity between baseline and follow up.

At baseline, cases spent significantly less time on productive activities in each country compared to controls (table 4). At follow-up, mean time spent on productive activities by operated cases increased compared to baseline in all three settings (p<0.001) (table 4). There was an average increase of 1hr 5mins (95% CI 0:21–1:47) in Kenya, 1hr 20mins (95%CI 0:40–1:59) in Bangladesh and 1hr 51mins (95%C I 0:50–2:52) in the Philippines (figure 1). In contrast among controls, time spent on productive activities either stayed the same (the Philippines) or decreased (Bangladesh and Kenya).

**Table 4:** Comparison of time spent on different activity categories at baseline and follow up among operated cases and controls.

		Kenya			Bangladesh			The Philippines		
Activity category	Case/control	BaselineMean time*hrs:mins (95%CI)	Follow upMean time*hrs:mins (95%CI)	P-value†	BaselineMean time*hrs:mins (95%CI)	Follow upMean time*hrs:mins (95%CI)	p-value†	BaselineMean time*hrs:mins (95%CI)	Follow upMean time*hrs:mins (95%CI)	p-value†
**Productive**										
	Operated cases	2:24(1:48–3:00)	3:30(2:54–4:12)	0.008	2:03(1:34–2:32)	3:26(2:47–4:05)	<0.001	3:42(2:57–4:27)	5:24(4:37–6:11)	0.001
	Controls	5:18(4:30–6:12)	4:36(3:48–5:24)	0.2	5:06(4:42–5:29)	4:29(4:04–4:54)	0.003	5:56(5:15–6:38)	6:01(5:24–6:39)	0.1
	p-value‡case vs control	<0.001	0.1		<0.001	0.03		<0.001	0.06	
**Leisure outside household**										
	Operated cases	0:21(0:12–0.31)	1:12(0:48–1:30)	<0.001	0:30(0:17–0:44)	1:13(0:59–1:28)	<0.001	0:59(0:40–1:18)	1:11(0:51–1:30)	0.3
	Controls	1:12(0:42–1:42)	1:30(1:06–1:54)	0.4	0:37(0:25–0:49)	1:04(0:54–1:15)	<0.001	1:04(0:46–1:22)	1:15(0:57–1:32)	0.3
	p-value‡case vs control	<0.001	0.3		0.7	0.3		0.6	0.7	
**Leisure inside household**										
	Operated cases	2:48(2:18–3:12)	3:30(3:00–4:00)	0.02	3:50(3:28–4:12)	3:56(3:34–4:17)	0.7	4:39(3:57–5:22)	3:58(3:28–4:29)	0.08
	Controls	3:06(2.36–3:30)	3:24(2:54–3:54)	0.3	3:22(3:11–3:34)	3:38(3:25–3:51)	0.04	3:07(2:42–3:32)	3:33(3:12–3:54)	0.05
	p-value‡case vs control	0.4	0.8		0.06	0.3		<0.001	0.3	
**No activity**										
	Operated cases	6:36(5:54–7:24)	4:30(4:06–5:00)	<0.001	5:20(4:48–5:52)	3:26(2:57–3:56)	<0.001	4:14(3:22–5:07)	4:05(3:18–4:52)	0.5
	Controls	4:00(3:24–4:42)	3:48(3:18–4:18)	0.6	3:06(2:48–3:24)	3:07(2:48–3:26)	0.9	4:33(3:50–5:15)	4:09(3:31–4:47)	0.4
	p-value‡case vs control	<0.001	0.04		<0.001	0.5		0.6	0.9	

* Mean time spent by participants with complete data at baseline and follow up. Participants with outlier values at either baseline or follow up were excluded.

† P-value from paired t-test comparing time-use at baseline and follow up.

‡ P-value from anova comparing time-use among operated cases versus controls at a) baseline and b)follow up, controlling for age and gender.

#### Leisure

At baseline, cases in Kenya spent significantly less time than controls on leisure outside of the household, while in the Philippines cases spent more time on leisure activities inside the household. At follow up, time spent on leisure outside of the household by operated cases increased in Kenya (from 21minutes (95% CI 0:12–0:31) to 1hrs 12mins (95% CI 0:48–1:30), p<0.001) and Bangladesh (30mins (95%CI 0:17–0:44) to 1hr13mins (95%Ci 0:59–1:28) p<0.001). However, a significant increase was also observed among controls in Bangladesh. In the Philippines, in contrast to the other two countries, time spent on leisure inside the home by operated cases decreased, though not significantly.

#### No activity

At baseline, in Kenya and Bangladesh, cases on average spent nearly two hours more in inactivity compared to controls (<0.001), while there was little difference in the Philippines. At follow-up there was a reduction of nearly two hours in average time spent on ‘no activity’ compared to baseline by operated cases in Kenya and Bangladesh (p<0.001, figure 1) with no change in the Philippines. Time spent on ‘no activity’ among controls did not change significantly from baseline and follow up.

### Assistance with activities at baseline and follow up

At baseline 26% (n = 26, Kenya), 43% (n = 43, Bangladesh) and 23% (n = 23, Philippines) of cases reported receiving assistance while this was lower for controls (n = 2, 2% Kenya; n = 20, 9% Bangladesh; n = 12, 9% Philippines, p<0.001). At follow up the proportion of operated cases reporting assistance with any activity during the previous day reduced by at least half compared to baseline in each country (table 3), although a decrease was also observed for un-operated cases in the Philippines (from n = 14, 15% to n = 5, 5%, p = 0.03, data not presented). Among controls, the proportion reporting receiving assistance increased in Bangladesh (from n = 20, 9% to n = 35, 16%, p = 0.03), but not in the other two countries. Most reported assistance was for personal activities (sleeping, bathing, dressing and eating).

### Comparison of cases and controls at follow up

After cataract surgery, time-use patterns of operated cases became more similar to controls (tables 3 and 4). The average time spent on productive activities by operated cases remained less than controls (approximately 1hour in Kenya and Bangladesh, 30mins in Philippines), although this difference was smaller than at baseline and not significant for most activity groups. In contrast, un-operated cases continued to spend between 2–3hrs less time (p<0.04), on productive activities and more time (1½–2hrs) doing ‘no activity’ compared to controls (p<0.003 in each country, data not presented).

### Predictors of change in time-use among operated cases

We explored the predictors of change in time spent on productive activities and inactivity from baseline to follow-up among the operated cases. Increases in time spent on productive activities were significantly larger among operated cases aged 50–60 years compared to those aged ≥80 years (p = 0.04, table 5) but no associations with other socio-demographic variables were found. People who were severely visually impaired (VA<6/60–3/60), blind (VA<3/60) or had perception of light at baseline had larger increases in time on productive activities compared to those with moderate visual impairment (VA <6/24–6/60) (p-for-trend = 0.001). Increases in time on productive activities were smaller among operated cases with poor VA outcome from surgery (VA<6/60) compared to those with good outcome (VA ≥6/18, p = 0.02). Change in time spent in inactivity was not associated with the socio-demographic and ocular factors included.

**Table 5:** Predictors of change in time spent on productive activities from baseline to follow up among operated cases across the three countries combined.

Variable		N	Mean change in time spent minutes (95% CI)	Coefficient (95% CI)*	p-value*
Age group (years)	50–59	28	197 (113–282)	Reference	
	69–69	73	82 (26–138)	−99.2 (−196.7– −1.6)	0.05
	70–79	108	56 (13–99)	−121.7 (−215.1–28.3)	0.01
	≥80	62	70 (23–116)	−109.3(−210.0– −8.6)	0.03
	*P for trend* *			0.07	
Gender	Male	122	79 (38–122)	Reference	
	Female	149	82 (47–117)	1.0 (4.8–166.1)	0.97
Baseline VA**	<6/24 – 6/60	82	8 (−38–55)	Reference	
	<6/60 – 3/60	104	104 (46–163)	85.5 (4.8–166.2)	0.04
	<3/60 >PL	88	88 (17–158)	89.5 (10.2–168.2)	0.03
	PL	130	130 (86–176)	121 (54.3–188.4)	<0.001
	*P for trend* *			0.001	
Follow up VA**	>6/18	205	97 (66–126)	Reference	
	6/18–6/60	41	51 (−23–126)	−55.0 (−130–20.9)	0.16
	<6/60	25	2 (−88–92)	−106 (−72.3–55.3)	0.02
	*P for trend* *			0.01	

*Coefficients, 95% Confidence Intervals and P-values are derived from the multivariate linear regression analysis.

**Presenting visual acuity in the better eye.

PL Perception of light.

There was no association between change in time spent and SES, literacy, marital status and unilateral/bilateral surgery and these data are not presented.

## Discussion

The Cataract Impact Study found that adults aged ≥50 years were more likely to undertake and spent significantly more time on productive activities after cataract surgery compared to before and this was consistent across three different low-income settings. Further, after surgery their time-use patterns become similar to those of age- gender-matched controls with normal vision. To our knowledge this is the first time the impact of cataract surgery has been explored through comparison of time-use patterns.

Time-spent on productive activities, after cataract surgery, increased by an average of one-two hours. Time spent in ‘inactivity’ reduced by nearly two hours in Kenya and Bangladesh and in the Philippines time spent on leisure inside the home decreased by nearly an hour. This corresponds with findings from baseline; in Kenya and Bangladesh at baseline, cases spent much more time “doing nothing” than controls while in the Philippines there were no differences in inactivity, but cases spent more time on leisure in the home. Reductions in both these activity groups may indicate a change towards increased engagement in more ‘active’ pastimes. The decreased proportion of operated cases reporting assistance at follow up, in each country, also indicates increased independence following cataract surgery.

Time use patterns can vary for a number of different reasons from one year to the next and it was to address this issue that control subjects were included in the study. The fact that increases in productive activities (engagement and time-spent) and reductions with reported assistance were consistently observed among operated cases, but not their age- gender matched controls lends important support to cataract surgery being the major causal factor in these changes among operated cases.

Many cultural and personal factors influence how people spend their time and it is neither possible nor desirable to define an ‘ideal’ time-use pattern. However the observed increase in productive activities supports the frequently postulated economic impact of cataract surgery. While this is most obvious for paid work, non-paid productive activities also make important economic contributions through different routes.[9] Where money for purchasing food is scarce, producing food for own use is an economic imperative. Domestic activities are essential components without which other activities contributing more directly to a household's economy cannot occur. Child-care undertaken by older adults may free up time for other household members to engage in income generating activities.[19] The reduced need for assistance with daily activities observed in this study after surgery may also contribute to this effect. Estimates from a UNDP report suggest that on a global level if “unpaid activities were treated as market transactions at the prevailing wages they would yield huge monetary valuations – a staggering $16 trillion”.[9] The increased participation in different productive activities observed in this study therefore provide some evidence of a mechanism by which cataract surgery may contribute to improved economy.

Increased engagement in daily activities and reduced inactivity and need for assistance also have important implications for well-being. Previous studies have shown a positive relationship between involvement in activities and well-being, cognitive function and life satisfaction among older adults. [7]–[8], [20] Further, these findings may indicate an increased role in family and community, supporting a human rights argument for sight restoration on the basis of improved inclusion in society.

The differences in time-use between operated cases and controls reduced compared to baseline. However, the overall time spent on productive activities by operated cases remained slightly lower at follow up, perhaps because of continued loss of functioning due to vision or other age-related conditions beside cataract or the difficulty in reversing a less active role in a household.

Limited research has been conducted in this area to allow comparison and no studies were identified that interviewed patients pre and post-operatively. Our findings support, to some extent, those of Javitt et al (1983) who found re-engagement in paid work and household activities following cataract surgery in India.[19] The reported proportion returning to paid work was much higher than in the current study (85% men, 58% women), perhaps because the study population was younger and the majority were in paid employment prior to sight loss.

The direct cost of cataract surgery to the patient in the study settings is between US$40–60 including follow up appointments and medicines. In each of these settings however, systems are in place to provide free surgery for patients who are unable to pay.

### Limitations

Limitations of the time-use data collection method include the reliance on recall of time-use and the collection of time-use for a single day, which may not be a ‘typical’ day. [10] Follow-up interviews were not always conducted in the same month as baseline and time of year may influence time-use patterns. It was not possible to mask interviewers as to the case/control status at baseline or follow up. There was no assessment of preferences regarding different activities and changes in time-use. We acknowledge that while there is a tendency to view productive activities as positive, the burden of too much work can also be a negative phenomenon. [21]. Increased participation in leisure outside of the home was observed for controls as well as operated cases in Bangladesh and the Philippines and therefore cannot be attributed to cataract surgery. This may be explained by timing of surveys in relation to community events (end of Eid during the baseline in Bangladesh and fiestas during follow up in the Philippines).

Simplified examination techniques were used to diagnose visual impairment from cataract and therefore co-morbidities contributing to poor vision may not have been detected. However this reflects the ‘real world’ situation where patients with co-morbidity commonly undergo surgery even if only small increases in vision are expected and, if anything, would likely result in smaller changes in time-use following cataract surgery. A substantial proportion of the cases did not attend for surgery in each country. Few socio-demographic differences between operated and un-operated cases were observed limiting the potential affect on external validity. The exception was that un-operated cases were older than the operated cases. It is therefore possible that if more of the un-operated cases had undergone surgery and been included at the follow up, average increases in time spent on productive activities may have been slightly smaller.

### Strengths

This was a large multi-country longitudinal study and similar trends in time-use change were seen across three countries. Cases and controls were population based, recruited from randomly selected clusters in the survey area (with the exception of 50 hospital cases in Kenya) and were therefore likely to be representative of the general population in the study areas. The relative stability of time-use among controls provides support to the reliability of the questionnaire and to cataract surgery being a major factor in the time-use changes observed among operated cases.

### Conclusion

In conclusion, this study provides empirical evidence of increased time spent on productive activities and less time in inactivity following cataract surgery among older adults in low-income settings. As well as the positive implications for well-being and inclusion, this provides support to arguments of economic gain from sight restoring cataract surgery.
